# Impact of an animal-assisted therapy programme on physiological and psychosocial variables of paediatric oncology patients

**DOI:** 10.1371/journal.pone.0194731

**Published:** 2018-04-04

**Authors:** Nathiana B. Silva, Flávia L. Osório

**Affiliations:** 1 Pio XII Foundation–Barretos Cancer Hospital, Barretos, São Paulo, Brazil; 2 Department of Neurosciences and Behavioural Sciences, Faculty of Medicine of Ribeirão Preto–USP, Ribeirão Preto, São Paulo, Brazil; Istituto Superiore Di Sanita, ITALY

## Abstract

The objective of this study was to propose an intervention and safety protocol for performing animal-assisted therapy (AAT) and evaluating its efficacy in children under outpatient oncological treatment based on psychological, physiological, and quality of life indicators for the children and caregivers. The sample consisted of 24 children diagnosed with leukaemia and solid tumours (58% girls with a mean age of 8.0 years) who underwent an AAT programme consisting of three 30-min sessions in an open group. Two dogs (one Labrador retriever and one golden retriever) were used, and activities such as sensory stimulation, gait training, and socialization were conducted. The exclusion criteria were severe mental problems, inability to answer the questions included in the instruments used, allergy to animals, unavailability/lack of interest, isolation precaution, surgical wound, use of invasive devices, ostomy, no current blood count for evaluation, neutropaenia, infection, fever, diarrhoea, vomiting, respiratory symptoms at the beginning of the intervention or 1 week before the intervention, hospitalization or scheduled surgery, and non-completion of the AAT programme. The variables analysed using validated self or other evaluations were stress, pain, mood, anxiety, depression, quality of life, heart rate, and blood pressure. A quasi-experimental study design was used. We observed a decrease in pain (p = 0.046, d = –0.894), irritation (p = 0.041, d = –0.917), and stress (p = 0.005; d = –1.404) and a tendency towards improvement of depressive symptoms (p = 0.069; d = –0.801). Among the caregivers, an improvement was observed in anxiety (p = 0.007, d = –1.312), mental confusion (p = 0.006, d = –1.350), and tension (p = 0.006, d = –1.361). Therefore, the selection criteria and care protocols used for the AAT programme in the oncological context were adequate, and the programme was effective.

## Introduction

Throughout their domestication process, animals have had great importance in human life, having been used for transport and hunting since ancient civilizations in all cultures. Furthermore, the biophilia hypothesis suggests that there is an innate human biological tendency to interact and form close connections and emotional bonds with other forms of natural life, especially with animals, starting in early childhood [1–4].

Human behaviour towards animals is complex and involves evolutionary, psychological, and cultural factors. The phylogenetic proximity and/or the physical, behavioural, and cognitive similarities between humans and other animals are only some of the factors that facilitate this relationship[5]. Other factors, such as the animal’s aesthetics, its anthropomorphic characteristics and its child-like morphological and behavioural traits, stand out among those that increase animals’ attractiveness to humans, especially to children, [6–8].

It is also worth noting that according to a study by Nagasawa et al. [9], companion animals, especially dogs, can contribute to the establishment of a human-animal bond that is behaviourally and neurohormonally similar to the mother-baby relationship. For these and other researchers, the psychological and psychophysiological effects associated with human-animal interactions also result from the activation of the oxytocinergic system and/or the facilitation of human-human relations (the so-called “social catalyst effect”) [9–10].

Recognition of the positive aspects of this mutual interspecies relationship has prompted some scholars to investigate the use of animals in promoting human health. Currently, the International Association of Animal-Interaction Organizations (IAHAIO) has standardized theoretical, practical, and ethical guidelines related to different forms of intervention involving human-animal interaction[11].

One of these techniques is Animal Assisted Therapy (AAT), which is “*a goal oriented*, *planned and structured therapeutic intervention directed and/or delivered by health*, *education and human service professionals*. *(…) AAT is delivered and/or directed by a formally trained (with active licensure*, *degree or equivalent) professional with expertise within the scope of the professionals’ practice*. *AAT focuses on enhancing physical*, *cognitive*, *behavioral and/or socio-emotional functioning of the particular human recipient*” [11] (p.5).

Scientific studies on AAT, particularly those involving dogs, have shown an openness to and an acceptance of this strategy by medical teams[12–18] and have documented recognition for AAT’s safety and efficacy in different environments and clinical contexts, including hospitalization[19–21], emergency medicine[17], oncology[22–29], cardiology[18,30], psychiatry[31–33], and outpatient and hospital paediatrics[34–38].

The results of studies on AAT are promising despite the lack of standardization of the number, duration, and frequency of sessions, the executed activities, and the safety measures for the animals and patients. Overall, the results indicate improvements in haemodynamic and physiological parameters[20,30] and decreases in the prescription of analgesics[21] and perceived pain[20]. Regarding psychosocial variables, there is evidence of improved mood[34] and reduced symptoms of depression and anxiety[19,20].

The use of AAT in the oncological context has been reported in several studies in adult populations[22–26,29]. These studies have indicated improvements in anxiety and stress and an increased sense of well-being in addition to providing a distraction in the hospital setting. Qualitative studies from a single research group involving children[27,28] have indicated an improvement in the acceptance of invasive procedures, improvement of the sense of well-being during hospitalization, reduction of depressive symptoms, and better adaptation to proposed therapies. However, no experimental quantitative studies have evaluated the effect of AAT in children to date.

In this context, the present study is a pioneer in the field of outpatient paediatric oncology in Brazil. The objectives of this study are a) to propose an intervention and safety protocol for the implementation of AAT and b) to evaluate the impact of an AAT programme in children undergoing outpatient oncological treatment based on psychological, physiological, and quality of life indicators for the children and caregivers.

## Materials and methods

This study used a quasi-experimental design[39] and was approved by the local ethics committees in animal research (Comitê de Ética no Uso de Animais do IRCAD América Latina—Process No. 054/2015) and human research (Comitê de Ética em Pesquisa–Fundação Pio XII–Hospital de Câncer de Barretos—Process No. 1009960).

### Participants

This convenience sample was not estimated statistically. The participants evaluated for eligibility for the study (n = 4983) were in outpatient care at a children’s oncology hospital from June 2015 to January 2017. Of these patients, 24 were selected because they met the inclusion and exclusion criteria.

The inclusion criteria were as follows: a) Both genders aged 6 to 12 years; Diagnosis of a solid tumour; b) Diagnosis of acute lymphoid leukaemia (in maintenance treatment in phase B of the RELLA B and RELLA T protocols, both starting at week 53 of maintenance B, and in the maintenance phase of the BFM 2002 protocol) ^[^^40^^,^^41^^]^; c) Undergoing outpatient oncological treatment; and d) Showing a good clinical status after evaluation, with authorization to participate in the study by the responsible physician.

The exclusion criteria were as follows: a) Children who were not interested in the intervention and/or were afraid of animals; b) Children with severe mental/cognitive problems that might lead to injuries/inconvenience to the animals and increase the likelihood of undesired events; c) Allergy to animals; d) Unavailability to participate in the sessions and/or nonattendance at the initial evaluation session; e) Inability to respond to the instruments; and f) Non-completion of the AAT programme.

In addition to these criteria, specific exclusion criteria were adopted for the children's participation in each session to increase the safety level of the study and to propose an eligibility protocol for intervention in this specific context; to the best of our knowledge, these criteria have not been described in detail in previous studies.

In this context, before participation in each AAT session, the children were evaluated by a nurse, and those who fit the following criteria were excluded: a) Isolation precaution; b) Presence of a surgical wound (suture, dehiscence, or drainage); c) Use of invasive devices (probe, catheter, or drain); d) Presence of ostomies; e) No current blood count available for evaluation; f) Neutropaenia (less than 500/mm^3^ or less than 1000/mm^3^ with likelihood of decreasing within the next 48 hours); g) Presence of severe infection, documented infection by resistant bacteria (*Staphylococcus aureus* or coagulase-negative oxacillin-resistant *S*. *aureus*, vancomycin-resistant *Enterococcus*, or cefepime-resistant and/or meropenem-resistant Gram-negative bacillus), or suspected or confirmed infection with *Clostridium difficile*; h) Fever, diarrhoea, vomiting, or respiratory symptoms during the week before the intervention; and i) Hospitalization or scheduled surgery.

**Fig 1:**
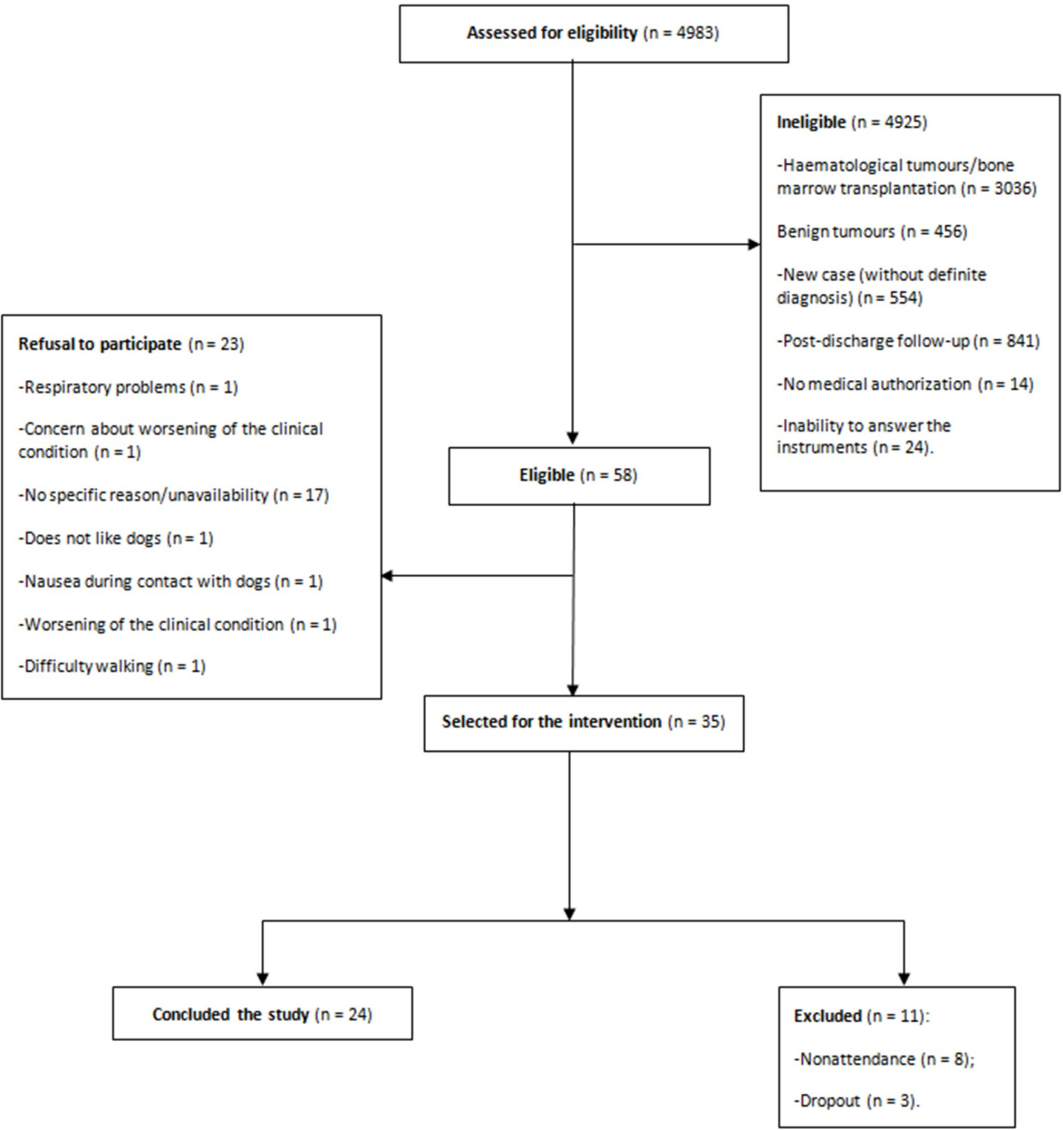
Flowchart of the inclusion and exclusion criteria.

The flowchart of the inclusion and exclusion criteria for the study participants is shown in Fig 1 (Fig 1).

A specific protocol for animal entry and handling in the hospital setting was created that complied with the guidelines for animal-assisted interventions in health care facilities[42]. The adopted parameters were as follows:

a) All individuals, whether targets of the intervention or members of the technical team, should wash their hands with water, soap, or alcohol every time they come into contact with the dogs;
b) The animals were temporarily suspended from activities if, within 7 days prior to the intervention, they presented with i) stress, fatigue, and/or aggression, ii) oestrus, immunodepression, infections, or open wounds, or iii) episodes of diarrhoea, vomiting, or urinary/faecal incontinence. In cases in which this last condition occurred during the session, the Hospital Infection Commission was consulted to provide care for the children who had come into contact with the animals;
c) In the event of a sphincter-related accident, the site should be properly cleaned, and the Hospital Infection Commission should be notified accordingly. In this situation, the animal should be re-educated to prevent the occurrence of such accidents;
d) Possible episodes of bites and/or scratches of the participants by the dogs should be reported to the medical and nursing staff and later to the Hospital Infection Commission, and hygiene and care measures should be provided by the whole team;
e) Prohibition of entry of the animal into specific hospital areas, such as kitchens and drug preparation areas. The animals were only allowed to enter the therapy room; and
f) Dog hygiene should also include bathing, hair brushing, nail trimming, flea and tick monitoring (for a minimum of 24 hours before the session), and disinfection/cleaning of the dog’s paws before and after the session with 70% alcohol.

### Animals and handler

One Labrador retriever and one golden retriever who were both healthy and carefully selected for this study were used for the intervention, because they met the following criteria: a) Docility, obedience training, and socialization; b) Approval of veterinary surgeons and animal behaviour specialists (trainers) after specific evaluation; c) Updated immunization records and anti-parasitic treatment; d) Quarterly clinical examinations (haematological and biochemical) by a veterinarian; e) Exclusive feeding on animal feed (dry food).

The handler was a physical therapist (NBA) with additional academic training in AAT who was also the owner of the animals. The animals and the handler had prior experience in this type of activity.

### Intervention

The AAT programme consisted of three 30-min sessions per week. The intervention occurred in an open group with a maximum of seven participants. The number of planned absences per participant was one, and the lost session needed to be replaced to complete a total of three sessions in a maximum of 4 weeks.

The activities carried out during the intervention were previously planned by the person delivering the intervention, who chose among the following per appropriateness for each session and depending on the interests and dynamics of the group: 1) sensorial and upper limb stimulation (brush, pet, and play fetch with the dog); 2) training on activities of daily living (give water and food to the food) and gait (walking with the dog); 3) socialization and recreation (dog show; playing with the dog’s supplies; dog drawing; agility courses—guide the dog through obstacles such as cones and ropes; dog clothes—form words that express feelings and attach them to the dog clothes with Velcro; stories about the dog—daily routine, origin).

### Instruments

The following self-applied instruments were used to evaluate outcome variables in the children:

a) Child Stress Symptoms Inventory: This instrument is composed of 35 items that evaluate the occurrence or not of stress in children. The instrument was proposed and validated by Lipp et al[43,44], and the original version was in Brazilian Portuguese. The items were self-administered, scored on a five-point Likert scale, and recorded in quarter circles according to the frequency with which the participants presented the symptoms described in each item as follows: never (0 points); rarely (1 point); sometimes (2 points); usually (3 points); and always (4 points). The maximum achievable score was 140 points.
b) Quality of Life Evaluation Scale: The instrument is used to evaluate the quality of life in children aged 4 to 17 years. It is composed of 26 questions that evaluate function, family, leisure, and autonomy. Each question is self-filled and scored from 0 (very unhappy) to 3 (very happy), and the maximum achievable score is 78. The instrument was originally proposed by Manificat and Dazord[45] and translated and validated for Brazilian Portuguese by Assumpção et al[46].
c) Child Depression Inventory: This self-administered instrument was designed for children aged 7 to 17 years to assess the presence and severity of depressive symptoms. It is composed of 20 items scored on a Likert scale from 0 to 2 points according to the symptoms presented in the past 2 weeks as follows: 0 (no symptoms); 1 (mild symptoms); and 2 (severe symptoms). The instrument was proposed by Kovacs[47], and the version adapted and validated for Brazilian Portuguese by Golfeto et al[48] was used.
d) Adapted Brunel Mood Scale: The adaptation of the Brunel Mood Scale (BRUMS) created by Terry and Lane[49] and validated for Brazilian Portuguese by Rohlfs et al[50] was used for the self-evaluation of the children's moods. Items 2, 6, 7, 10, 11, 12, 13, 14, 15, 19, 20, 21, and 22 were used and scored by the participants using a Likert scale ranging from 0 (not at all) to 4 (extremely).
e) Faces Pain Scale: This self-applied instrument is used to measure the intensity of perceived pain in children. It is composed of six horizontal faces expressing different degrees of pain. The child chooses the face that best represents his/her degree of pain. The instrument was proposed by Hicks et al[51] and translated and validated for Brazilian Portuguese by Silva and Thuler[52].
f) AAT Assessment Questionnaire: This instrument was prepared by the authors to evaluate the participants' impressions of the AAT programme. It consisted of five questions, namely: i) *"Did you like playing with the dog in the hospital*?; ii) *Do you think that the activities with the dog helped your treatment?*; iii) *What did you think about spending time with the dog?*; iv) *Do you think it would be nice to always have a dog in the hospital?*; and v) *Would you suggest this activity with the dog to your friends?*.

The following instruments were used to evaluate the outcomes in caregivers:

a) State-Trait Anxiety Inventory: This other-evaluation instrument is used to quantify subjective components related to anxiety. It consists of 34 items that describe children's behaviour or problems. The items were scored by parents or guardians according to the child's degree of suffering on a scale ranging from 0 (absent) to 3 (always). The instrument was developed by Spielberger[53] and adapted and validated for Brazilian Portuguese by Silva et al[54].
b) Brunel Mood Scale: This self-applied tool was originally developed by Terry and Lane[39] and validated for Brazilian Portuguese by Rohlfs[50]. It is composed of 24 items scored using a five-point Likert scale ranging from 0 (not at all) to 4 (extremely). This instrument was completed by the caregivers, who answered the items according to the child's mood.
c) Sociodemographic and Clinical Identification Questionnaire: This instrument was constructed for the present study to collect data on the sociodemographic and clinical characteristics of the sample.

Additionally, the blood pressure and heart rate were measured using the BP3ABOH-G-Tech semi-automatic pressure meter appropriate for the participant’s age.

### Data collection

Initially, the nursing team contacted the eligible patients and subsequently their caregivers after evaluation of the children by the responsible physician. Children who met the inclusion and exclusion criteria and their parents signed consent and assent forms and were included in the study. Before the start of the first AAT session, the participants and their caregivers completed the instruments described above with the help of a member of the research team trained for this purpose. The heart rate and blood pressure were also measured. At the end of the programme (i.e., end of the third session), the participants and their caregivers filled out the same instruments again, and the physiological parameters were re-measured.

### Data analysis

The data were manually entered into a database and analysed using the Statistical Package for the Social Sciences software (IBM SPSS Statistics 18). Sociodemographic and clinical characteristics were analysed using descriptive statistics. The evaluations before and after the AAT programme were compared using non-parametric statistics with the Wilcoxon test. The size of the effect of the differences within groups was calculated using Cohen’s d, with four sizes being described as follows[55]: small (≤ 0.2); medium (0.2–0.5); large (0.5–1.0); and very large (> 1). A level of significance of p < 0.05 was considered for all analyses.

## Results

The sociodemographic and clinical characteristics of the study sample are shown in Table 1.

**Table 1:** Sociodemographic and clinical characteristics of the study sample.

Variables	N	%
***Sex***		
*Female*	14	58.3
*Male*	10	41.7
***Age***		
*Mean (SD)*	8.58 (1.98)	
***Education***		
*Illiterate*	4	16.7
*Can read and write*	5	20.8
*Incomplete elementary education*	15	62.5
***Duration of therapy***		
*Mean (SD)–months*	11.2 (16.7)	
*Median–months*	8.5	
*Minimum/Maximum–months*	1/65	
***Treatment period***		
*≤ 30 days*	6	24.7
*> 30 days*	18	75.3

SD = standard deviation; N = number of individuals in the sample; % = percentage of the sample

The sample had a higher predominance of girls (58%), with a mean age of 8.58 years (range: 6 to 12 years). The most common types of tumours were Ewing's sarcoma (20.8%) and rhabdomyosarcoma (12.4%). In addition, 16.4% of the children presented allergies, but this condition did not prevent their participation in the programme, and none of the evaluated children had severe mental or cognitive problems.

Tables 2 and 3 show the results of the studied variables before and after the AAT programme.

**Table 2:** The children’s clinical and physiological indicators before and after completion of the AAT programme (n = 24).

Variables	Pre-AAT Mean (SD)	Post-AAT Mean (SD)	P-value^(A)^	*Effect Size* (d)
***Heart rate (bpm)***	103.42 (16.32)	109.42 (18.51)	0.121	0.668
***Blood pressure (mmHg)***				
*Minimum*	63.75 (8.93)	61.04 (12.91)	0.153	–0.624
*Maximum*	97.92 (9.55)	95.57 (18.19)	0.573	–0.237
***Presence of pain***				
*Faces Pain Scale*	0.41 (1.01)	0.08 (0.40)	0.046*	–0.894
***Mood (adapted from the BRUMS)***				
*Lively*	3.38 (0.87)	3.58 (0.83)	0.248	0.485
*Downhearted*	0.21 (0.58)	0.04 (0.20)	0.194	–0.550
*Annoyed*	0.50 (1.18)	0 (0)	0.041*	–0.917
*Sleepy*	0.54 (1.02)	0.63 (1.09)	0.951	0.025
*Angry*	0.33 (0.96)	0.13 (0.61)	0.102	–0.707
*Miserable*	0.17 (0.38)	0.04 (0.20)	0.180	–0.570
*Anxious*	3.12 (1.51)	2.71 (1.65)	0.373	–0.370
*Worried*	0.46 (0.93)	0.50 (1.25)	0.887	0.058
*Active*	3.00 (1.41)	2.75 (1.70)	0.584	–0.225
*Angry*	0.17 (0.63)	0.13 (0.44)	0.854	–0.075
*Energetic*	3.50 (1.02)	3.04 (1.39)	0.209	–0.531
*Tired*	0.42 (0.83)	0.50 (0.88)	0.762	0.124
*Bad-tempered*	0.25 (0.67)	0.08 (0.40)	0.336	–0.401
***Child Stress (ESI)***	38.29 (17.75)	30.25 (14.75)	0.005*	–1.404
***Quality of life (AUQEI)***	53.63 (6.96)	52.88 (8.00)	0.558	0.241
*Autonomy*	10.88 (3.17)	10.92 (2.45)	0.895	0.053
*Leisure*	15.13 (2.25)	14.88 (1.96)	0.692	–0.162
*Function*	12.25 (3.31)	12.33 (2.91)	0.835	0.085
*Family*	15.38 (2.41)	14.75 (2.94)	0.206	–0.535
***Depression (CDI)***	10.13 (5.64)	8.08 (4.85)	0.069	–0.801

AUQEI = Quality of Life Evaluation Scale; BRUMS = Brunel Mood Scale, adapted; CDI = Child Depression Inventory

(A) Wilcoxon's test

SD = standard deviation; ESI = Child Stress Symptoms Inventory

(d) sample size

*Significant difference

bpm = beats per minute; mmHg = millimetre of mercury.

**Table 3:** The caregivers’ clinical indicators before and after completion of the AAT programme (n = 24).

Variables	Pre-AAT	Post-AAT	P-value ^(A)^	*Effect size* (d)
	Mean (SD)	Mean (SD)		
***Anxiety (STAI)***	32.04 (16.51)	26.04 (11.85)	0.007*	–1.312
***Mood (BRUMS—Subscales)***				
*Anger*	3.38 (4.16)	2.08 (3.56)	0.080	–0.764
*Mental confusion*	3.54 (3.36)	1.71 (2.24)	0.006*	–1.350
*Fatigue*	2.42 (3.26)	1.21 (1.86)	0.136	–0.639
*Tension*	4.75 (3.76)	2.21 (2.06)	0.006*	–1.361
*Depression*	2.17 (3.51)	0.83 (1.58)	0.077	–0.774
*Vigour*	9.83 (3.50)	10.38 (3.05)	0.726	0.144

BRUMS = Brunel Mood Scale; SD = standard deviation; STAI = State-Trait Anxiety Inventory

*Significant difference

(A) Wilcoxon's test

(d) sample size.

Among the children, significant improvement was observed in the pain levels (p = 0.046, d = –0.894), irritation (p = 0.041, d = –0.917), and stress (p = 0.005, d = –1.404), and a tendency towards a decrease was observed for depressive symptoms (p = 0.069; d = –0.801) with large or very large effect sizes. No significant changes in the physiological indicators were observed.

Among the caregivers, significant improvement was observed in the indicators of the Brunel Mood Scale, including anxiety (p = 0.007; d = –1,312), stress (p = 0.006; d = –1.350), and mental confusion (p = 0.006; d = –1.361) (the latter included the items uncertain, muddled, anxious, and worried), and the effect size was classified as very large. There was a tendency towards a decrease in the rate of depression (p = 0.077; d = –0.774). These data are shown in Table 3.

Notably, no complications were observed during the study period for the patients and dogs, and no association was found between worsening of the clinical status of the children and the proposed therapy according to the medical evaluation. Furthermore, the therapy dogs did not show any signs of stress during the study.

## Discussion

AAT is an innovative therapeutic modality that has a positive impact on different psychological and physiological variables independent of the target public and the therapeutic context (hospitalized adults[19–21], emergency medicine[17], oncology[22–29], cardiology[30], psychiatry[31–33], and hospital paediatrics[34–38]). An example is better adaptation to the hospital setting and a reduction in stress, anxiety, depressive symptoms, and cortisol levels[56–62].

Therefore, there has been high acceptance of this therapy as a coadjuvant strategy for the management of different medical conditions by medical teams in recent years. Paediatric oncology is inserted into this context, because the diagnosis and treatment of childhood cancer causes physical and emotional suffering for children and increases their vulnerability to the development of psychological disorders, which may directly or indirectly affect their general clinical condition.

Previous studies in Canada have described pilot procedures and the results of an AAT programme for children with cancer[27,28]. This programme was designed to assist children younger than 15 years of age under treatment for haematological and solid tumours. The interventions lasted 12 months, and children who attended the AAT sessions could not present allergies, severe neutropaenia, recent surgery, use of invasive devices, or aggressive behaviour. The authors reported that the benefits of AAT in this context were evidenced by the qualitative perception/evaluation by the caregivers and hospital staff, including improved adaptation to the hospital setting, increased appetite, pain relief, acceptance of invasive procedures, increased sensation of well-being, less suffering, and increased motivation. Considering the positive indicators reported by this research group and the absence of quantitative studies in this context, the objective of the present study was to measure the effects of AAT on psychosocial and physiological parameters of paediatric oncology patients.

The first challenge and a factor that set this study apart was the proposal of a systematic protocol with clear and rigorous inclusion and exclusion criteria for the target patients of the therapy as well as the care to be taken in terms of the hospital environment and therapy animals. This protocol was developed in cooperation with a nosocomial infection control team specialized in oncology. The programme had high acceptance by the hospital staff as a whole, and its execution was feasible. Moreover, no complications that could endanger the health of the patients and animals were observed.

These findings corroborate those of previous studies, which reported the safety of the use of AAT in hospitals when care was taken in the indication and inclusion of patients in the intervention[14,27–29,34,36–39,63,64].

Consistent with the findings of previous studies, our results indicated improvement in pain and psychological parameters (irritation, stress, anxiety, mental confusion, and tension) of the children undergoing outpatient oncological treatment as perceived by the caregivers and children, with significant effect sizes.

The improvement observed after participation in three AAT sessions may be directly related to the benefits of the human-animal relationship, which favours psychological and endocrine changes in the human body. Previous studies have shown that visual communication and touching animals can trigger the release of various substances in the human body, including oxytocin, endorphins, and serotonin, and reduce the baseline cortisol level[48,49,65–74]. These hormones and cytokines in combination may contribute to a reduction of pain, anxiety, and stress and increase the sensation of pleasure and relaxation by children undergoing cancer treatment.

These results can also be explained from the perspective of interspecies relations, because there is no competition and instead there is a type of mutualism that justifies the increase in communication and social relations. The formation of emotional bonds and ties with animals makes their company enjoyable, especially during treatment, and can fill some challenges faced by children during daily oncological treatment, including greater distance from relatives, friends, and school activities and fewer social interactions[54,75].

The distraction provided by AAT is notable [i.e., the change in the focus of attention from the disease (symptoms, procedures, limitations, and restrictions) to health]. AAT was more effective than other leisure activities used in the hospital setting, such as reading, interaction with volunteers, and recreational activities[22,23,25,30,70], probably due to the specificities of the affiliative and affective relationship between dogs and humans.

In contrast to the results of previous studies, the symptoms of depression did not improve significantly in our study despite the tendency towards a reduction in the symptoms compared to the baseline levels. This result may be because the follow-up period was not sufficient to allow capture of significant changes in this and the other mood and physiological parameters analysed; additionally, the sample size was small, which weakened the power of the statistical tests used and increased the likelihood of type II error (i.e., not identifying differences).

In conclusion, the proposed programme was effective for children in outpatient oncological treatment considering the quantitative effect on the analysed variables, and the selection criteria and safety precautions adopted for the participants, dogs, and hospital environment were adequate considering the acceptance of the programme by the medical team and the lack of complications.

However, this study has the following limitations: 1) a quasi-experimental design^64^ and the absence of a control group and 2) a relatively small sample, which was not statistically estimated and was restricted to specific types of tumours.

Therefore, more studies are necessary to address these limitations and produce robust evidence that can prove the effectiveness of AAT in promoting the physical, mental, and emotional well-being of children undergoing oncological treatment and the humanization of the hospital environment.

## References

[1] DeCourcey M, Russel AC, Keister KJ. Animal- Assisted Therapy: Evaluation and Implementation of a Complementary Therapy to Improve the Psychological and Psysiological Health of Critically III Patients. Dimens Crit Care Nurs 2010, 29 (5): 211–214. doi: 10.1097/DCC.0b013e3181e6c71a 20703127 10.1097/DCC.0b013e3181e6c71a

[2] Willens JS. Animal- assisted therapies are becoming more common. Pain Manag Nurs 2013, 14 (4): 183 doi: 10.1016/j.pmn.2013.10.001 24315240 10.1016/j.pmn.2013.10.001

[3] Antonioli C, Reveley MA. Randomised controlled trial of animal facilitated therapy with dolphins in the treatment of depression. BMJ 2005, 331(7527):1231 doi: 10.1136/bmj.331.7527.1231 16308382 10.1136/bmj.331.7527.1231 PMC1289317

[4] Borgi M, Cirulli F. Pet Face: Mechanisms Underlying Human-Animal Relationships. Front Psychol 2016, 7:298 doi: 10.3389/fpsyg.2016.00298 27014120 10.3389/fpsyg.2016.00298 PMC4782005

[5] Serpell J. Factors influencing human attitudes to animals and their welfare. Anim Welfare 2004, 13:145–151.

[6] Stokes D. Things we like: human preferences among similar organisms and implications for conservation. Hum Ecol 2007, 35: 361–369.

[7] Lišková S, Frynta D. What determines bird beauty in human eyes? Anthrozoös 2013, 26: 27–41.

[8] Borgi M, Cogliati-Dezza I, Brelsford V, Meints K, Cirulli F. Baby schema in human and animal faces induces cuteness perception and gaze allocation in children. Front Psychol 2014, 5:411 doi: 10.3389/fpsyg.2014.00411 24847305 10.3389/fpsyg.2014.00411 PMC4019884

[9] Nagasawa M, Mitsui S, En S, Ohtani N, Ohta M, Sakuma Y et al Social evolution. Oxytocin- gaze positive loop and the coevolution of human-dog bonds. Science 2015, 348: 333–336. doi: 10.1126/science.1261022 25883356 10.1126/science.1261022

[10] Beetz A, Uvnäs-Moberg K, Julius H, Kotrschal K. Psychosocial and psychophysiological effects of human-animal interactions: the possible role of oxytocin. Front Psychol. 2012, 3:234 doi: 10.3389/fpsyg.2012.00234 22866043 10.3389/fpsyg.2012.00234 PMC3408111

[11] Jegatheesan B, Beetz A, Ormerod E, Johnson R, Fine A, Yamazaki K et al The IAHAIO White Paper: Definitions for Animal Assisted Intervention and Guidelines for Wellness of Animals Involved. 2014, 1:1–10. Available from: http://iahaio.org/wp/wp-content/uploads/2017/05/iahaio-white-paper-final-nov-24-2014.pdf

[12] Eaglin V. Attitudes and Perceptions of Nurses-in-Training and Psychiatry and Pediatric Residents towards Animal-Assisted Interventions. Hawaii Med J. 2008, 67 (2): 45–47. 18438089

[13] Moody WJ, Maps RK, Rourke S. Attitudes of paediatric medical ward staff to a dog visitation programme. J Clin Nurs. 2002; 11 (4): 537–544. 12100650 10.1046/j.1365-2702.2002.00618.x

[14] Wu AS, Niedra R, Pendergast L, McCrindle BW. Acceptability and Impact of Pet Visitation on a Pediatric Cardiology Inpatient Unit. J Pediatr Nurs. 2002; 17 (5): 354–362. 12395303 10.1053/jpdn.2002.127173

[15] Stoffel JM, Braun CA. Animal-Assisted Therapy: Analysis of Patient Testimonials. [Internet] [accessed in apr 10 2014]. Available from: http://juns.nursing.arizona.edu/articles/Fall%202006/stoffel.htm.

[16] Bibbo J. Staff Members Perceptions of an Animal-Assisted Activity. Oncol Nurs Forum. 2013; 40 (4): 320–326. 10.1188/13.ONF.E320-E326 23803276

[17] Nahm N, Lubin J, Lubin J, Bankwitz BK, Castelaz M, Chen X, et al Therapy dogs in the emergency department. West J Emerg Med. 2012; XIII (4): 363–365. 10.5811/westjem.2011.5.6574 PMC3421977 22942937

[18] Novotny NL, Deibner J, Herrmann C. Animal-assisted therapy to promote ambulation in the hospital setting: Potentially effective but is it feasible? J Nurs Educ Pract. 2015; 5 (7): 123–130.

[19] Hoffmann AOM, Lee AH, Wertenauer F, Ricken R, Jansen JJ, Gallinat J, et al Dog-assisted intervention significantly reduces anxiety in hospitalized patients with major depression. Eur J Integr Med. 2009: 145–148.

[20] Nepps P, Stewart CN, Bruckno SR. Animal-assisted activity: Effects of a complementary intervention program on psychological and physiological variables. J Evid Based Complementary Altern Med. 2014; 19 (3): 211–215. doi: 10.1177/2156587214533570 24789913 10.1177/2156587214533570

[21] Havey J, Vlasses FR, Vlasses PH, Ludwig-Beymer P, Hackbarth D. The effect of Animal-assisted therapy on pain medication use after joint replacement. *Anthrozoös*. 2014; 27 (3): 361–369.

[22] Johnson RA, Meadows RL, Haubner JS, Sevedge K. Human-animal interaction: A complementary/alternative medical (CAM) intervention for cancer patients. Am Behav Sci. 2003; 47 (1): 55–69.

[23] Johnson RA, Meadows RL, Haubner JS, Sevedge K. Animal-Assisted Activity among patients with cancer: Effects on mood, fatigue, self-perceived health, and sense of coherence. Oncol Nurs Forum. 2008; 35 (2): 225–232. doi: 10.1188/08.ONF.225-232 18321834 10.1188/08.ONF.225-232

[24] Fleishman SB, Homel P, Chen MR, Rosenwald V, Abolencia V, Gerber J, et al Beneficial effects of animal-assisted visits on quality of life during multimodal radiation-chemotherapy regimens. J Community Support Oncol. 2013; 13 (1): 22–26. 10.12788/jcso.0102 25839062

[25] Marcus DA, Blazek-O´Neill B, Kopar JL. Symptom reduction identified after offering Animal-Assisted Activity at a cancer infusion center. AJHPM. 2014; 31 (4): 420–421. 10.1177/1049909113492373 23728415

[26] White JH, Quinn M, Garland S, Dirkse D, Wiebe P, Hermann M, et al Animal-assisted therapy and counseling support for women with breast cancer: Na exploration of patient´s perceptions. Integr Cancer Ther. 2015; 14 (5): 460–467. doi: 10.1177/1534735415580678 25897045 10.1177/1534735415580678

[27] Gagnon J, Bouchard F, Landry M, Belles-Isles M, Fortier M, Fillion L. Implementing a hospital-based animal therapy program for children with cancer: A descriptive study. Can Oncol Nurs J. 2004; 14 (4): 217–22. 15635895 10.5737/1181912x144217222

[28] Bouchard F, Landry M, Belles-Isles M, Gagnon J. A magical dream: A pilot project in animal-assisted therapy in pediatric oncology. Can Oncol Nurs J. 2004: 14–17. 15040146 10.5737/1181912x1411417

[29] Orlandi M, Trangeled K, Mambrini A, Tagliani M, Ferrarini A, Zanett L, et al Pet Therapy Effects on Oncological Day Hospital Patients Undergoing Chemotherapy Treatment. Anticancer Res. 2007; 27 (6C): 4301–4304. 18214035

[30] Cole KM, Gawlinski A, Steers N, Kotlerman J. Animal-Assisted Therapy in Patients Hospitalized With Heart Failure. Am J Crit Care. 2007; 16 (6): 575–585. 17962502

[31] Aoki K, Iwahashi K, Ishigooka J, Fumihiko F, Numajiri M, Ohtani N, et al Evaluation of cerebral activity in the pré-frontal córtex in mood [affective] disorders during animal-assisted therapy (AAT) by near-infrared scpectroscopy (NIRS): A pilot study. Int J Psychiatry Clin Pract. 2012; 16 (3): 205–213. doi: 10.3109/13651501.2011.644565 22486555 10.3109/13651501.2011.644565

[32] Berget B, Ekeberg O, Pedersen I, Braastad BO. Animal- assisted therapy with farm animals for persons with psychiatric disorders. Ann Ist Super Sanita. 2011; 27 (1): 50–64. 10.4415/ANN_11_04_10 22194073

[33] Berget B, Ekeberg O, Braastad BO. Animal-assisted therapy with farm animals for persons with psychiatric disorders: effects on self-efficacy, coping ability and quality of life, a randomized controlled trial. Clin Pract Epidemiol Ment Health. 2008; 4 (9): 1–7. 18405352 10.1186/1745-0179-4-9 PMC2323374

[34] Kaminski M, Pellino T, Wish J. Play and Pets: The Physical and Emotional Impact of Child- Life and Pet Therapy on Hospitalized Children. Child Health Care. 2002; 31 (4): 321–335.

[35] Caprilli S, Messeri A. Animal- Assisted Activity at A. Meyer Children´s Hospital: A Pilot Study. eCAM. 2006; 3 (3): 379–383. doi: 10.1093/ecam/nel029 16951723 10.1093/ecam/nel029 PMC1513141

[36] Sobo EJ, Eng B, Kassity-Krich N. Canine Visitations (Pet) Therapy: Pilot Data on Decreases in Child Pain Perception. J Holist Nurs. 2006; 24 (1): 51–57. doi: 10.1177/0898010105280112 16449747 10.1177/0898010105280112

[37] Braun C, Stangler T, Narveson J, Pettingell S. Animal-assisted therapy as a pain relief intervention for children. Complement Ther Clin Pract. 2009; 15: 105–109. doi: 10.1016/j.ctcp.2009.02.008 19341990 10.1016/j.ctcp.2009.02.008

[38] Calcaterra V, Veggiotti P, Palestrini C, Giorgis V, Raschetti R, Tumminelli M, et al Post-operative benefits of Animal-assisted therapy in pediatric surgery: A randomised study. PLoSOne. 2015; 10 (6): 1–13. 10.1371/journal.pone.0125813 PMC4454536 26039494

[39] Harris AD, McGregor JC, Perencevich EM, Furuno JP, Zhu J, Peterson DE, et al The use and interpretation of quasi-experimental studies in medical informatics. AMIA. 2006; 13 (1): 16–23. 10.1197/jamia.M1749 PMC1380192 16221933

[40] Riviera GK, Ribeiro RC. Improving treatment of children with acute lymphoblastic leukemia in Developing countries through technology sharing, collaboration, and partnerships. Expert Rev Hematol. 2014; 7 (5): 649–657. doi: 10.1586/17474086.2014.949233 25174644 10.1586/17474086.2014.949233 PMC4174393

[41] Aricó M, Valsecchi MG, Conter V, Rizzari C, Pession A, Messina C. et al Improved outcome in high-risk childhood acute lymphoblastic leukemia defined by prednisone-poor response treated with double Berlin-Frankfurt-Muenster protocol II. Blood. 2002; 100 (2): 420–426. 12091331 10.1182/blood.v100.2.420

[42] Lefebvre SL, Golab GC, Christensen E, Castrodale L, Aureden K, Bialachowski A, et al Guidelines for animal-assisted interventions in health care facilities. Am J Infect Control. 2008; 36 (2): 78–85. doi: 10.1016/j.ajic.2007.09.005 18313508 10.1016/j.ajic.2007.09.005

[43] Lipp MEN, Lucarelli MDM (1998). Escala de Stress Infantil (ESI). São Paulo: Casa do Psicólogo; 1998.

[44] Lipp MEN, Arantes JP, Buriti MS, Witzig T. O estresse em escolares. Psicol Esc Educ. 2002; 6 (1): 51–56.

[45] Manificat S., Dazord A. Évaluation de la qualité de vie de l’enfant: validation d’un questionnaire, premiers résultats. Neuropsychiatr Enfance Adolesc. 1997; 45: 106–114.

[46] Assumpção FBJ, Kuczynski E, Sprovieri MH, Aranha EMG. Escala de avaliação de qualidade de vida (AUQEI- AUTOQUESTIONNAIRE QUALITÉ DE VIE ENFANT IMAGÉ)- Validade e confiabilidade de uma escala para qualidade de vida em crianças de 4 a 12 anos. Arq Neuropsiquiatr. 2000; 58 (1): 119–127. 10770876 10.1590/s0004-282x2000000100018

[47] Kovacs M. The Children’s Depression Inventory (CDI). Psychopharmacol Bull. 1985; 21 (4): 995–998. 4089116

[48] Golfeto JH, Veiga MH, Souza L, Barbeira C. Propriedades psicométricas do Inventário da Depressão Infantil (CDI) aplicado em uma amostra de escolares de Ribeirão Preto. Rev Psiq Clin. 2002; 29 (2): 66–70.

[49] Terry PC, Lane AM, Fogarty GJ. Construct validity of the POMS-A for use with adults. Psychology of Sport and Exercise. 2003; 4: 125–39.

[50] Rohlfs ICPM, Rotta TM, Luft CB, Andrade A, Krebs RJ, Carvalho T. A Escala de Humor de Brunel (BRUMS): Instrumento para Deteccao Precoce da Síndrome do Excesso de Treinamento- Brunel Mood Scale (BRUMS): an Instrument for Early Detection of Overtraining Syndrome. Rev Bras Med Esporte. 2008; 14 (3): 176–181.

[51] Hicks CL, Von Baeyer CL, Spafford PA, Van Korlaar I, Goodenough B. The Faces Pain Scale-Revised: toward acommon metric in pediatric pain measurement. Pain. 2001; 93: 173–83. 11427329 10.1016/S0304-3959(01)00314-1

[52] Silva FC, Thuler LCS. Cross-cultural adaptation and translation of two pain assessment tools in children and adolescentes- Tradução e adaptação transcultural de duas escalas para avaliação da dor em crianças e adolescentes. Jornal de Pediatria. 2008; 84 (4): 344–349. doi: doi:10.2223/JPED.1809 18688551 10.2223/JPED.1809

[53] Spielberger CD. Manual for the State—Trait Anxiety Inventory for Children. Palo alto, CA: Consulting Psychologist Press: 1973.

[54] Silva WV, Figueiredo VLM. Ansiedade infantil e instrumentos de avaliação: uma revisão sistemática- Childhood anxiety and assessment instruments: a systematic review. Rev Bras Psiquiatr. 2005; 27 (4): 329–35. 16358117 10.1590/s1516-44462005000400014

[55] Maroco J. Análise Estatística–Com Utilização do SPSS. Lisboa: Edições Sílabo; 2007.

[56] Redd R, Ferrer L, Villegas N. Natural curators: a review of therapy and animal-assisted activities as complementary treatment of chronic diseases. Rev Lat Am Enfermagem. 2012; 20 (3): 1–7. 10.1590/s0104-11692012000300025 22991126

[57] Lasa SM, Bocanegra NM, Alcaide RV, Arratibel MAA, Donoso EV, Ferriero G. Animal assisted interventions in neurorehabilitation: a review of the most recente literature. Neurologia. 2015; 30 (1): 1–7. doi: 10.1016/j.nrl.2013.01.012 23642347 10.1016/j.nrl.2013.01.012

[58] O´Haire ME, Guérin NA, Kirkham AC. Animal-assisted intervention for trauma: a systematic literature review. Front Psychol. 2015; 6 (1121): 1–13. 26300817 10.3389/fpsyg.2015.01121 PMC4528099

[59] Engelman S. Palliative care and use of Animal-assisted therapy. Omega. 2013; 67 (1–2): 63–67. doi: 10.2190/OM.67.1-2.g 23977780 10.2190/OM.67.1-2.g

[60] Beetz A, Uvnas-Moberg K, Julius H, Kotrschal K. Psychosocial and psychophysiological effects of human-animal interactions: the possible role of oxytocin. Front Psychol. 2012; 3 (234): 1–15. 22866043 10.3389/fpsyg.2012.00234 PMC3408111

[61] Krause-Parello CA, Friedmann E. The effects of an Animal-assisted intervention on salivar alpha-amylase, salivar immunoglobulin A, and heart rate during forensic interviews in child sexual abuse cases. *Anthrozoös*. 2014; 27 (4): 581–590.

[62] Barker SB, Knisely JS, Schubert CM, Grren JD, Ameringer S. The effect of an Animal- Assisted Intervention on anxiety and pain in Hospitalized Children. *Anthrozoös*. 2015; 28 (1): 101–112.

[63] Tsai C, Friedmann E, Thomas AS. The effect of Animal Assisted Therapy on stress responses in Hospitalized children. *Anthrozoös*. 2010; 23 (3): 245–258.

[64] DeCourcey M, Russel AC, Keister KJ. Animal- Assisted Therapy: Evaluation and Implementation of a Complementary Therapy to Improve the Psychological and Psysiological Health of Critically III Patients. Dimens Crit Care Nurs. 2010; 29 (5): 211–2014. doi: 10.1097/DCC.0b013e3181e6c71a 20703127 10.1097/DCC.0b013e3181e6c71a

[65] Trembath F MPH. Animal-Assisted Intervention for People with Cancer. Habri Central. 2015; (17): 1–5.

[66] Odendaal JSJ. Animal-assisted therapy–magic or medicine? J Psychosom Res. 2005; 49: 275–280. 10.1016/s0022-3999(00)00183-5 11119784

[67] Takashima GK, Day MJ. Setting the one health agenda and the human-companion animal bond. International Int J Environ Res Public Health. 2014; 11: 11110–11120. 10.3390/ijerph111111110 PMC4245602 25350006

[68] Stewart LA, Dispenza F, Parker L, Chang CY, Cunnien T. A pilot study assessing the effectiveness of na Animal-assisted outreach program. JMH. 2014; 9 (3): 332–345.

[69] Barker S, Knisely JS, McCain NL, Best AM. Measuring stress and imune response in healthcare professional following interaction with a therapy dog: A pilot study. Psychol Rep. 2005; (96): 713–719. 16050629 10.2466/pr0.96.3.713-729

[70] Motooka M, Koike H, Yokoyama T, Kennedy NL. Effect of dog-walking on autonomic nervous activity in sênior citizens. MJA. 2006; 184 (2): 60–63. 16411869 10.5694/j.1326-5377.2006.tb00116.x

[71] Vormbrock JK, Grossberg JM. Cardiovascular Effects of Human-Pet Dog Interactions. J Behav Med. 1988; 11 (5): 509–517. 3236382 10.1007/BF00844843

[72] Goddard AT, Gilmer MJ. The Role and Impact of Animals with Pediatric Patients. CNE. 2015; 41 (2): 65–71. 26292453

[73] Carine S, Ades C. Benefícios que o convívio com um animal de estimação pode promover para saúde e bem-estar do ser humano In: Chelini MOM, Otta E, editors. Terapia Assistida por Animais. São Paulo: Editora Manole; 2016 p. 37–40.

[74] Albuquerque NS, Ciari MB. Cães e seres humanos: uma relação forte, complexa, duradoura e vantajosa In: Chelini MOM, Otta E, editors. Terapia Assistida por Animais. São Paulo: Editora Manole; 2016 p. 18–20.

[75] Faraco CB. Human-Dog interaction: the social constituted by the interspecies relation. Thesis (Doctorate). Porto Alegre: Pontifical Catholic University of Rio Grande do Sul; 2008.

